# Effect of Orthokeratology on myopia progression: twelve-year results of a retrospective cohort study

**DOI:** 10.1186/s12886-017-0639-4

**Published:** 2017-12-08

**Authors:** Yueh-Chang Lee, Jen-Hung Wang, Cheng-Jen Chiu

**Affiliations:** 1 Department of Ophthalmology, Buddhist Tzu Chi General Hospital, No. 707, Sec. 3, Zhongyang Rd, Hualien City, 97002 Taiwan; 2 Department of Medical Research, Buddhist Tzu Chi General Hospital, Hualien, Taiwan; 3 Institute of Medical Sciences, Tzu Chi University, Hualien, Taiwan; 4 Department of Ophthalmology and Visual Science, Tzu Chi University, Hualien, Taiwan

**Keywords:** Myopia, Myopia control, Orthokeratology, Contact lenses, Optical intervention

## Abstract

**Background:**

Several studies reported the efficacy of orthokeratology for myopia control. Somehow, there is limited publication with follow-up longer than 3 years. This study aims to research whether overnight orthokeratology influences the progression rate of the manifest refractive error of myopic children in a longer follow-up period (up to 12 years). And if changes in progression rate are found, to investigate the relationship between refractive changes and different baseline factors, including refraction error, wearing age and lens replacement frequency. In addition, this study collects long-term safety profile of overnight orthokeratology.

**Methods:**

This is a retrospective study of sixty-six school-age children who received overnight orthokeratology correction between January 1998 and December 2013. Thirty-six subjects whose baseline age and refractive error matched with those in the orthokeratology group were selected to form control group. These subjects were followed up at least for 12 months. Manifest refractions, cycloplegic refractions, uncorrected and best-corrected visual acuities, power vector of astigmatism, corneal curvature, and lens replacement frequency were obtained for analysis.

**Results:**

Data of 203 eyes were derived from 66 orthokeratology subjects (31 males and 35 females) and 36 control subjects (22 males and 14 females) enrolled in this study. Their wearing ages ranged from 7 years to 16 years (mean ± SE, 11.72 ± 0.18 years). The follow-up time ranged from 1 year to 13 years (mean ± SE, 6.32 ± 0.15 years). At baseline, their myopia ranged from −0.5 D to −8.0 D (mean ± SE, −3.70 ± 0.12 D), and astigmatism ranged from 0 D to −3.0 D (mean ± SE, −0.55 ± 0.05 D). Comparing with control group, orthokeratology group had a significantly (*p* < 0.001) lower trend of refractive error change during the follow-up periods. According to the analysis results of GEE model, greater power of astigmatism was found to be associated with increased change of refractive error during follow-up years.

**Conclusions:**

Overnight orthokeratology was effective in slowing myopia progression over a twelve-year follow-up period and demonstrated a clinically acceptable safety profile. Initial higher astigmatism power was found to be associated with increased change of refractive error during follow-up years.

## Background

Myopia is the most common refractive disorder, [1, 2] it is also one of the leading causes of visual impairment worldwide [3, 4]. The prevalence of myopia varies geographically, for example, 30% of the population in Australia [5] and North American [6], and up to 85% of the East Asia population, especially in Taiwan [7, 8]. Among myopic patients, high myopia is especially associated with increased risk of ocular comorbidities, [9, 10] increased socioeconomic burden, [3, 4, 11, 12] and compromised quality of life [13, 14]. Therefore, the prevention of myopia progression is a major public health issue [8].

Many interventions have been developed to suppress the progression of myopia, including pharmaceutical agents (for example, atropine [15] and pirennzepine [16–18]), bifocal lenses [19, 20], multifocal lenses, [21, 22] aberration control spectacle lenses, [23] and soft and gas-permeable contact lenses [24–29]. Topical atropine reduces myopia progression and axial elongation in children in a dose-related manner. However, it has been reported to bring adverse effects, such as allergy, photophobia and a rebound phenomenon occur with higher doses [30, 31].

The concept of orthokeratology (OK) was first introduced in the 1950s by Wesley and Jessen as spectacle blur, a phenomenon describing corneal reshaping after wearing hard contact lenses. For its material was poor at oxygen permeation, making long-term wearing infeasible, orthokeratology was more of a novelty back then. In the 1970s, rigid gas permeable lenses improved comfort and safety by allowing more oxygen permeability. However, the lenses still remained incapable of effectively correcting myopia until the first reverse geometry lens designed by Richard Wlodyga introduced in 1989, which improved lens centration and myopia correction from −1 diopters (D) to −1.7 D. Up to the present, improvement of orthokeratology mainly involves using higher Dk lens material, different reverse geometry lens designs, and advances in corneal topography [32].

By the reverse geometry design of orthokeratology lens, the lens molds the cornea of a myopic eye into plateau shape. These orthokeratology lenses have much flatter central base curve than the secondary curve, thus create positive pushing pressure against the central cornea and negative pulling pressure against the mid-peripheral cornea, redistributing the epithelial cells to the mid-periphery while flattening the central cornea via a thinning of the epithelial layer. Through plateau-shaped cornea, light would be refracted simultaneously onto the mid-peripheral retina and macula, leaving the peripheral retina with relative myopic defocus [33–35]. Hyperopic peripheral defocus on the contrary, often found in myopic children, is believed to encourage eye growth. Manipulation of peripheral defocus toward myopia is hypothesized to stabilize eye growth and reduce myopia progression. Several studies reported its efficacy for myopia control by slowing axial elongation of the eyeball [36–38] and has been confirmed in a two-year randomized clinical trial [39].

Somehow, there is limited publication with follow-up longer than 3 years. A five-year prospective study that assessed the efficacy of OK showed a reduced rate of manifest refraction progression in OK-wearing eyes in the first 3 year of treatment [40]. Two retrospective studies comparing children wearing OK lenses with single-vision spectacles showed a reduced rate of manifest refraction progression in OK-wearing eyes over 7-year and 8-year periods [41, 42].

This retrospective study aims to investigate whether overnight OK influences the progression rate of the manifest refractive error of myopic children in a longer follow-up period (up to 12 years), and to investigate the relationship between refractive changes and different baseline factors, including refraction error, wearing age and lens replacement frequency. In addition, this study collects long-term safety profile of overnight OK.

## Methods

This is a retrospective study of sixty-six school-age children who visited a private ophthalmology clinic between January 1998 and December 2013, and received overnight OK correction. Sixty-four school-age children wearing spectacles were identified from the same practice, and 36 subjects whose baseline age and refractive error matched with those in the OK group were selected to form control group. A single practitioner (CJC) performed all examinations of the OK and spectacle wearers. This study was conducted in accordance with the tenets of the Declaration of Helsinki and approved by the Institutional Ethical Committee Review Board (REC No.: IRB103–17-B).

The subjects included in this study must meet the following criteria: 1) below 18 years of age 2) spherical refractive error less than −8.0 D 3) cylinder refractive error less than −3.0 D 4) distant best-corrected visual acuity (BCVA) better than 0 log minimum angle of resolution (logMAR) units (20/20) 5) follow-up period greater than 12 months. Subjects with underlying ocular disease such as retinopathy, prematurity, neonatal problems, history of genetic disease, neurodevelopment condition that might affect refractive development, or connective tissue disorders associated myopia were excluded from this study. Enrolled subjects could not have any amount of tropia by cover-uncover test at far (4.0 m) and near (0.33 m). All subjects underwent comprehensive examination including manifest refraction, cycloplegic refraction, uncorrected visual acuity (UCVA), best-corrected visual acuity, extraocular movements, intraocular pressure, slit-lamp examination and dilated fundoscopy at the first visit. Manifest refraction and cycloplegic refraction following instillation of two drops of 1% tropicamide were obtained with autorefractor (Speedy-K, Nikon, Tokyo, Japan). Snellen visual acuity chart was placed 6 m from the patient, and the white background of the chart was illuminated to 85 ± 5 cd/m^2^.

An accelerated OK reverse geometry design lens (Dreimlens®; Macro Vision Corp., Taiwan) was used in this study. The nominal central thickness of the lenses was 0.22 mm and the diameter was 10.4 to 11.2 mm. The material used in the overnight OK was a fluorosilicone acrylate material with nominal oxygen permeability (Dk) of 127 × 10^−11^ cm^2^/s (mL O_2_/mL mmHg). The same practitioner (CJC) fitted all the OK lenses using standardized fitting criterion.

After baseline examination, each patient underwent a 2-h daytime trial before the first night of lens wearing to confirm a successful orthokeratology contact lens fit. If an acceptable contact lens fit and fluorescein pattern were established, subjects were instructed to wear their contact lenses for at least 6–8 consecutive hours every night. The recommended daily cleaning protocol is first cleaning lens with Boston Advanced Cleaner and Conditioning Solutions (Bausch & Lomb), then rinsing the lenses with a sterile, preserved saline solution.

Clinical follow-up interval was 2–3 months on average. In follow-up examinations, UCVA and findings of slit-lamp examination of each visit were collected. Autorefraction and keratometry were performed using the Speedy-K autorefractor, cycloplegic refractions were measured every year. To minimize any potential acuity regression, all the examinations were completed before 11 AM within 2 hours after contact lens removal. All instruments were calibrated before study initiation.

Refraction over lens, BCVA and lens inspection to assess lens condition were also collected at each visit. Changes to manifest refractive error were calculated indirectly by determining the change in treatment curve (back optic zone radius, BOZR) required to maintain a refraction over lens (ROL) of plano. To ensure the OK eyes were not over-treated, all lenses were designed to have achieved a ROL of plano at baseline. If ROL of −0.5 D was found, this would suggest an increase in the myopia compared with baseline. Based on the fitting principles, the BOZR would require flattening 0.1 mm to restore a full refractive treatment [43]. The lenses were replaced every 1 to 2 years on average in order to maintain the wearing quality. Stable lens replacement frequency was defined as average replacement period less than 2 years.

We recorded and compared the change of visual acuity and the change in corneal curvature, manifest refractive error by ROL method at baseline and at the 1-year to 12-year follow-up. The manifest refractive error was expressed as spherical and cylindrical power, or power vectors: M represents the spherical equivalent of a refraction, J0 represents the astigmatism power vector associated with a horizontally oriented axis, J45 corresponds to the astigmatism power vector associated with an obliquely oriented axis in the 45° meridian [44, 45]. The data were expressed as frequencies, proportions, or means ± standard error of the mean, depending on the characteristics of each item. Some basic statistical tests were performed to compare the difference between groups. An independent t test was used to compare the means of continuous variables between different groups, whereas a paired t test was used to compare change of measurement results for paired samples. A Chi-squared test or Fisher Exact test was used to examine differences with categorical variables. Since correlated data from paired-eyes and repeated measurements across time were collected in this longitudinal study, the generalized estimating equations (GEE) was adopted to compare the results during the years to evaluate the association between the outcome and risk factors [46]. Statistically significant differences were defined as *p* < 0.05. All of the statistical analyses were performed using SPSS software version 17.0 (SPSS Inc., Chicago, IL, USA).

## Results

**Fig. 1:**
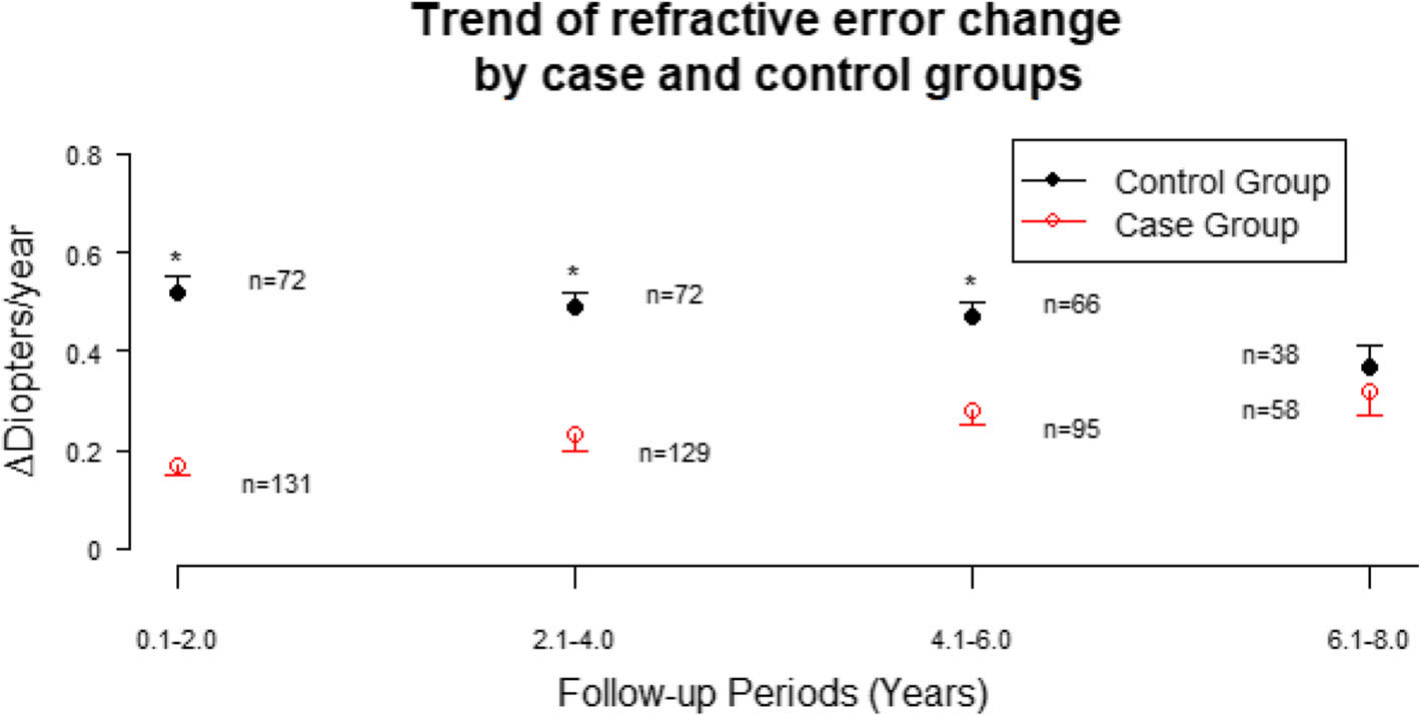
Trend of refractive error change. The trend of refractive error change during year 0 to year 2 for OK and control groups were 0.17 ± 0.02 D and 0.52 ± 0.03 D (mean ± SE), respectfully. The trend of refractive error change during year 2 to year 4 for OK and control groups were 0.23 ± 0.03 D and 0.50 ± 0.03 D (mean ± SE), respectfully. The trend of refractive error change during year 4 to year 6 for OK and control groups were 0.28 ± 0.03 D and 0.47 ± 0.03 D (mean ± SE), respectfully. The trend of refractive error change during year 6 to year 8 for OK and control groups were 0.32 ± 0.05 D and 0.37 ± 0.04 D (mean ± SE), respectfully. Asterisk (*) denotes statistically significant difference between OK and control groups

Data of 203 eyes were derived from 66 OK subjects (31 males and 35 females) and 36 control subjects (22 males and 14 females). Baseline demographics and ocular characteristics were summarized in Table 1, there were no significant differences between the control and OK groups. Their wearing ages ranged from 7 years to 16 years (mean ± SE, 11.72 ± 0.18 years). The follow-up time ranged from 1 year to 12 years (mean ± SE, 6.32 ± 0.15 years). At baseline, their myopia ranged from −0.5 D to −8.0 D (mean ± SE, −3.70 ± 0.12 D), and astigmatism ranged from 0 D to −3.0 D (mean ± SE, −0.55 ± 0.05 D). The quantities of M, J0, and J45 were −3.54 ± 0.14, −0.26 ± 0.03, and −0.03 ± 0.01 (mean ± SE) respectively. The overall trend of refractive error change per two-year period of OK and control groups were shown in Fig. 1. Compared with control group, OK group had a significantly (*p* < 0.001) lower refractive error change during the follow-up period. Furthermore, characteristics of OK group were classified into two subgroups according to its extent or range and were summarized in Table 2. About 70.2% of OK subjects started orthokeratology corrections at the age more than 10 years old. About 30.5% of OK subjects were with myopia more than −5.0 D. About 11.2% of subjects were with astigmatism greater than or equal to −1.5 D. About 82.4% of OK subjects were wearing with stable lens replacement frequency, which was defined as average replacement period less than 2 years.

**Table 1:** Comparison of Demographics between OK Group and Control Group

Variable	OK Group	Control Group	*P*-value	Total
Number of eyes	131	72		203
Wearing Age	11.65 ± 0.24	11.83 ± 0.26	0.628	11.72 ± 0.18
Gender			0.058	
Male	61 (46.6%)	44 (61.1%)		105 (51.7%)
Female	70 (53.4%)	28 (38.9%)		98 (48.3%)
Sphere refraction (D)	−3.82 ± 0.14	−3.48 ± 0.20	0.167	−3.70 ± 0.12
Cylinder refraction (D)	−0.56 ± 0.07	−0.52 ± 0.08	0.683	−0.55 ± 0.05
M	−3.54 ± 0.14	−3.22 ± 0.19		−3.43 ± 0.12
J0	−0.26 ± 0.03	−0.25 ± 0.04		−0.26 ± 0.03
J45	−0.03 ± 0.01	0.03 ± 0.01		−0.01 ± 0.01
Follow-up Time (years)	6.20 ± 0.21	6.53 ± 0.17	0.291	6.32 ± 0.15

Data are presented as n(%) or mean ± standard error of the mean

*p-value < 0.05 was considered statistically significant after test

D, diopters; M, the spherical equivalent; J0, the astigmatism power vector associated with a horizontally oriented axis; J45, the astigmatism power vector associated with an obliquely oriented axis in the 45° meridian

**Table 2:** Baseline Characteristics and Treatment Parameters of 66 OK subjects

Variable	n(%) or mean ± S.E.
Number of eyes	131
Gender	
Male	61(46.6%)
Female	70 (53.4%)
Age	11.65 ± 0.24
< =10 years-old	39 (29.8%)
> 10 years-old	92 (70.2%)
Ocular sphere refraction (D)	−3.82 ± 0.14
< −5D	91 (69.5%)
> = − 5D	40 (30.5%)
Ocular cylinder refraction (D)	−0.56 ± 0.07
< −1.5D	117 (89.3%)
> =1.5D	14 (10.7%)
M	−3.54 ± 0.14
J0	−0.26 ± 0.03
J45	−0.03 ± 0.01
Keratometry value (D)	43.64 ± 0.11
Follow-up time (years)	6.20 ± 0.21
Regular lens replacement	
No	23 (17.6%)
Yes	108 (82.4%)

Data are presented as n(%) or mean ± standard error of the mean

D, diopters; M, the spherical equivalent; J0, the astigmatism power vector associated with a horizontally oriented axis; J45, the astigmatism power vector associated with an obliquely oriented axis in the 45° meridian

**Fig. 2:**
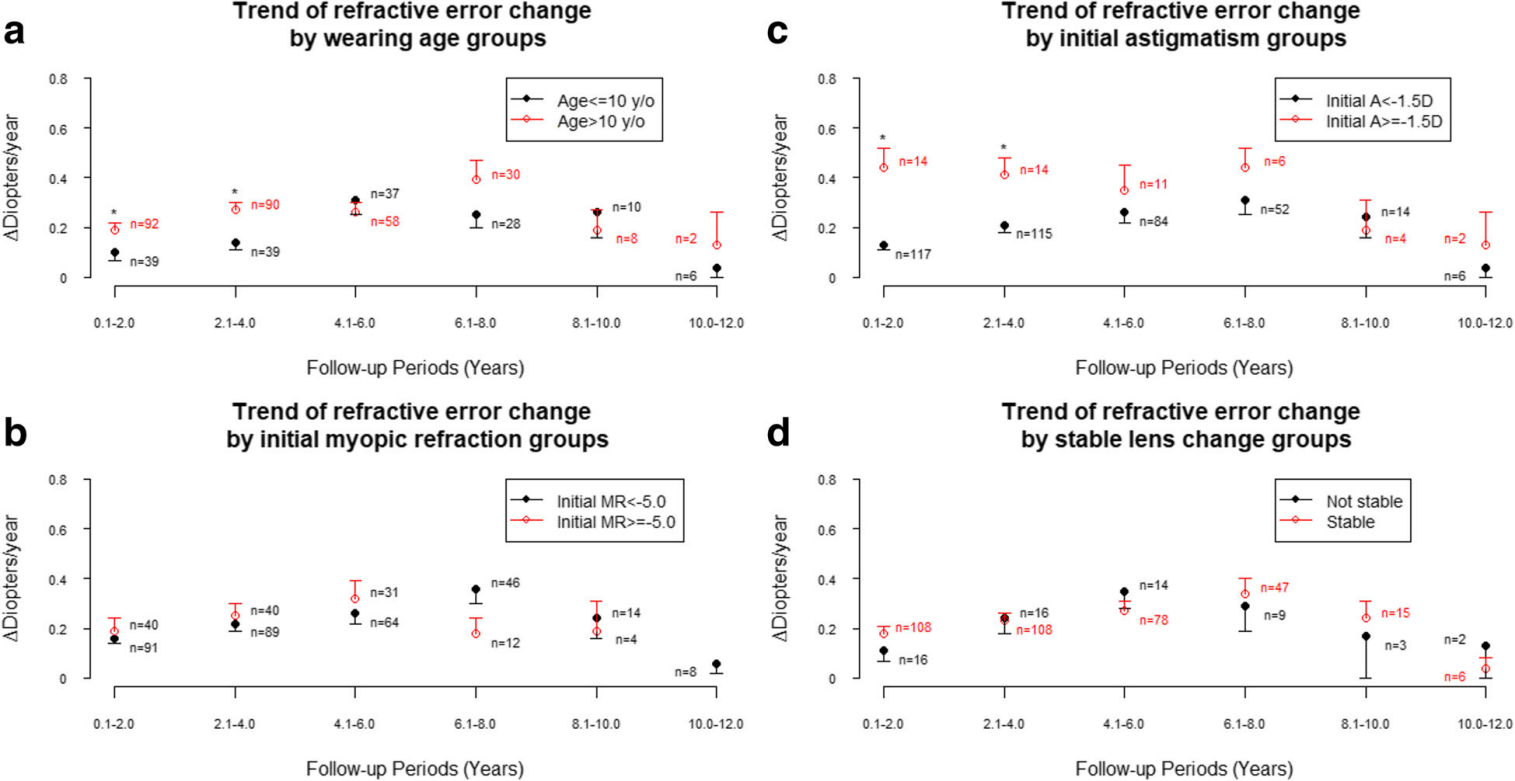
Trend of refractive error change (**a**) By different wearing age groups (**b**) By initial myopic refraction groups (**c**) By initial astigmatism groups and (**d**) By stable lens change groups. Asterisk (*) denotes statistically significant difference between OK and control groups

Further analysis was performed for OK subjects to evaluate the progression rate of the manifest refractive changes and of the association between the manifest refractive changes and baseline factors. The effect of orthokeratology corrections on change of corneal curvature per two-year period decreased gradually in the follow-up years (Table 3). There was decreased follow-up rate after the first 4 years, around only 14% of subjects remained in the study after 8 years. Therefore, statistical analysis of evaluating the association between the manifest refractive change and risk factors was performed for both follow up periods, including first 8 years and total 12 years. According to the analysis results of GEE model (Tables 4 and 5), astigmatism greater than or equal to −1.5 D and greater J0 (more negative) were found to be associated with increased change of refractive error during follow-up years. The trends of refractive error change per two-year period for different wearing age groups all showed that the change increased gradually in the first few years then started to decrease afterwards (Fig. 2a). Subjects with wearing age more than 10 years old tend to have larger refractive error change than those with wearing age less than 10 years old during first 4 years, but the overall difference between two groups was not significant in GEE model. The trends of refractive error change per two-year period for different initial myopic refraction groups all showed that the change increased gradually in the first few years then started to decrease afterwards (Fig. 2b). The overall difference between two groups was also not significant in GEE model. The trends of refractive error change per two-year period for different initial astigmatism groups showed significant difference (Fig. 2c). Subjects with astigmatism greater than or equal to −1.5 D tend to have larger increase of refractive error change during follow-up years (*p* = 0.007, Fig. 2c). The overall difference between two groups was significant in both GEE models. The trends of refractive error change per two-year period for stable and unstable lens replacement groups showed that the change increased gradually in the first few years then started to decrease afterwards (Fig. 2d). The overall difference between two groups was not significant in GEE model.

**Table 3:** The effect of orthokeratology corrections at different periods over 12 years follow-up

Item	1st to 2nd year	3rd to 4th year	5th to 6th year	7th to 8th year	9th to 10th year	11th to 12th year
Number of eyes	131	129	95	58	18	8
Change of refractive error	0.17 ± 0.02	0.23 ± 0.03	0.28 ± 0.03	0.32 ± 0.05	0.23 ± 0.07	0.06 ± 0.04
Change of corneal curvature	−1.72 ± 0.08	−1.57 ± 0.09	−1.45 ± 0.11	−1.45 ± 0.16	−1.18 ± 0.30	−2.14 ± 0.69

Data are presented as n or mean ± standard error of the mean

**Table 4:** GEE Model predicting change of refractive error over 8 years follow-up. (*n* = 131)

Predictor	β	S.E.	Z	*p* value
A				
Intercept	−0.039	0.105	−0.37	0.711
Wearing Age(>10 y/o vs. <=10 y/o)	0.063	0.051	1.24	0.215
Stable Change(Yes vs. No)	−0.014	0.010	−1.43	0.151
Initial D(> = −5D vs. <−5D)	−0.012	0.054	−0.22	0.829
Initial A(> = −1.5D vs. <−1.5D)	0.181	0.070	2.60	0.009*
B				
Intercept	−0.039	0.105	−0.37	0.711
Wearing Age (>10 y/o vs. <=10 y/o)	0.063	0.051	1.24	0.215
Stable Change (Yes vs. No)	−0.014	0.010	−1.43	0.151
M	−0.012	0.010	−1.14	0.255
J0	−0.189	0.051	−3.72	<0.001*
J45	−0.076	0.099	−0.77	0.444

Abbreviation: GEE, generalized estimating equations

*p-value < 0.05 was considered statistically significant after test

D, diopters; M, the spherical equivalent; J0, the astigmatism power vector associated with a horizontally oriented axis; J45, the astigmatism power vector associated with an obliquely oriented axis in the 45° meridian

**Table 5:** GEE Model predicting change of refractive error over 12 years follow-up. (*n* = 131)

Predictor	β	S.E.	Z	*p* value
a				
Intercept	−0.010	0.091	−0.11	0.916
Wearing Age(>10 y/o vs. <=10 y/o)	0.058	0.050	1.17	0.240
Stable Change(Yes vs. No)	−0.013	0.010	−1.38	0.167
Initial D(> = −5D vs. <−5D)	−0.007	0.053	−0.13	0.898
Initial A(> = −1.5D vs. <−1.5D)	0.153	0.056	2.75	0.006*
b				
Intercept	−0.010	0.091	−0.11	0.916
Wearing Age (>10 y/o vs. <=10 y/o)	0.058	0.050	1.17	0.240
Stable Change (Yes vs. No)	−0.013	0.010	−1.38	0.167
M	−0.013	0.010	−1.38	0.169
J0	−0.182	0.045	−4.02	<0.001*
J45	−0.045	0.102	−0.44	0.659

Abbreviation: GEE, generalized estimating equations

*p-value < 0.05 was considered statistically significant after test

D, diopters; M, the spherical equivalent; J0, the astigmatism power vector associated with a horizontally oriented axis; J45, the astigmatism power vector associated with an obliquely oriented axis in the 45° meridian

During the study period, mild superficial punctate keratopathy was observed in 8 subjects and mild corneal erosion was noted in 2 subjects. But these events recovered completely after discontinuation of lens wear for 1 to 2 weeks, and lens re-design in one subject. No other severe complications, such as corneal ulcer, were found in these OK users.

## Discussion

In this retrospective study with long term follow-up period (up to 12 years) conducted to investigate whether where overnight OK influences the progression of manifest refractive error in myopic children, OK group had a significantly (*p* < 0.001) lower refractive error change during the follow-up period. The refractive error change of OK group was around 0.2 to ~0.3 diopters per /year, while those wearing single-vision glasses had 0.4 to ~0.5 diopters per /year. Reviewing prior publications, published literature, the rate of myopia progression in our control group is comparable to other studies regarding myopic control using by orthokeratology [35–38]. Although previous reports demonstrate that overnight OK can retard the rate of myopia progression, there is limited published data beyond 3 years of follow-up. Our results revealed myopia control effect by orthokeratology could up to 8 years, with lessening the differences between the two groups gradually. ROMIO study [39] reported a time dependent apparent reduced efficacy on myopic control using OK, which was also observed in Hiraoka et al.'s results [40]. In their opinion, the reduction resulted from the gradual slowing of myopic progression in the control group with age, which was confirmed as a natural process, instead of reduced OK efficacy. This finding was also reported in prior studies [36–39, 47]. In a five-year prospective study by Hiaroka et al., they reported no additional beneficial effect for retarding myopia progression using orthokeratology after 3 years of lens wear [45]. However, children tend to have slower myopia progression with age, which was confirmed by previous studies. In meta-analysis by Donovan et al., myopia progression was faster in younger children, with greatest change of myopia in Chinese children reported ranging in age from 9 to 11 years [46]. Our results showed better myopia control by orthokeratology in the first 8 years of the study period, with the differences between the two groups narrowing gradually. The reduced myopic control effect may due to decreasing progression as age increased. This finding was also reported in prior studies [35–38].

Our study utilized standard lens design (spherical 4-zone lens), this limited the result applied only to children with low-to-moderate myopia and low astigmatism and who could achieve satisfactory orthokeratology response. Nowadays, different lens designs aiming to improve the performance of orthokeratology lenses became available, such as toric orthokeratology designs [40, 47]. It is expected orthokeratology for myopic control now applicable to a wider range of population in terms of children with higher degrees of myopia and astigmatism, thereby allowing more children to benefit from the myopic control treatment using orthokeratology. Many studies have been conducted to test if the performance of orthokeratology lenses to reduce myopia regression improved by new lens designs [48].

In our analysis of the relationship between refractive power change and different baseline refraction error, wearing age and lens replacement frequency, to evaluate the effect of orthokeratology corrections and its association between characteristics, only initial astigmatism power was found to be associated with change of refractive error progression during 12 years of follow -up years. The higher degrees of initial astigmatism, the greater progression of refractive error overmyopia refraction power with time. As with-the-rule astigmatism is the most common astigmatism in childhood, we found the absolute value of J0 was greater than J45 in both OK and control groups. Both high astigmatism (> − 1.5 D) and greater J0 (more negative) were found to be associated with increased change of refractive error during follow-up years. We hypothesize the lack of toric orthokeratology lens for high astigmatism may lead to incomplete myopic defocus of peripheral retina, thereby offset the effect of retarding myopia progression.The lack of toric orthokeratology lens for high astigmatism may led to incomplete myopic defocus of peripheral retina, thereby offset the effect of retarding myopia progression. In addition, the disparity of case number between children with high astigmatism power and those with less astigmatism power, may have made the statistical result unstable.

One limitation of our study is that myopia progression was measured by change of refraction over lens but not axial length, thus the reported differences in myopic refractive change was not necessarily correlated to real differences in ocular growth. Somehow, the same approach was used in Downie et al.’s study with reported advantages of its simple and all-embracing nature [42]. Thus, we confer the finding of change of refraction over lens in this study is related to myopic progression, not just a temporary effect. There is another limitation that tropicamide was used as cycloplegic agent instead of cyclopentolate. A previous study has shown 1% tropicamide is an effective cycloplegic agent for myopic children [48]. Cycloplegic refraction using 1% tropicamide is a proven method and was used in published study [49]. We also acknowledge the retrospective design of this study might carry the potential bias. Moreover, the disparity of case number between high astigmatism and low astigmatism may have made the statistical result unstable [41]. Besides, the retrospective design of this study might also carry the potential for both practitioner and investigator bias as neither the examiner nor the patient were masked to the treatment groups. Only standard-design lens (spherical 4-zone lens) was available during our study period limited the result applied only to children with low-to-moderate myopia and low astigmatism and who could achieve satisfactory orthokeratology response. Nowadays, different lens designs aiming to improve the performance of orthokeratology lenses became available, such as toric orthokeratology designs [50, 51]. Orthokeratology now is more applicable to a wider range of population with higher degrees of myopia and astigmatism. Besides, the optimal treatment duration and ideal starting age remain unclear. Our further clinical studies will try to clarify these issues.

## Conclusions

In conclusion, our study showed that OK treatment was effective in slowing progression of myopic refractive error over a twelve-year treatment period and demonstrated a clinically acceptable safety profile in a population of patients aged seven to sixteen years. Initial astigmatism power was the essential influential factor of change in refractive error during follow-up years. Initial wearing age, initial myopic power and lens replacement were found to have no effect on progression of myopic refractive error.

## Abbreviations

BCVA: Best-corrected visual acuity
D: Diopters
GEE: Generalized estimating eqs.
J0: the astigmatism power vector associated with a horizontally oriented axis
J45: the astigmatism power vector associated with an obliquely oriented axis in the 45° meridian
logMAR: log minimum angle of resolution
M: the spherical equivalent
OK: Orthokeratology
ROL: Refraction over lens
UCVA: Uncorrected visual acuity
